# Genetic  Diversity of the BLV *env* Gene and gp51 Mutations in Genotypes G4 and G7 Circulating in Dairy Cattle in the Novosibirsk Region (Western Siberia, Russia)

**DOI:** 10.3390/pathogens15040405

**Published:** 2026-04-08

**Authors:** Dmitry Baboshko, Kirill Elfimov, Polina Achigecheva, Irina Osipova, Grigoriy Vlasov, Oleg Rozhkov, Boyko Margarita, Aleksey Totmenin, Aleksandr Agaphonov, Natalya Gashnikova

**Affiliations:** 1 State Research Center of Virology and Biotechnology “Vector”, 630559 Koltsovo, Russianmgashnikova@gmail.com (N.G.); 2 Razdolnoye LLC, 632654 Kochenevo, Russia; 3 GBU NSO “Novosibirsk Veterinary Directorate”, 630007 Novosibirsk, Russia; 4 Faculty of Natural Sciences, Novosibirsk State University, 630090 Novosibirsk, Russia

**Keywords:** bovine leukemia virus, BLV, genetic diversity of BLV, phylogenetic analysis, recombination analysis, mutation analysis, Novosibirsk region, Russia

## Abstract

Bovine leukemia virus (BLV) is an oncogenic retrovirus and the etiological agent of enzootic bovine leukosis (EBL), which is spread worldwide. This study presents data on the genetic diversity of BLV in the Novosibirsk region of Russia. ELISA-positive samples were selected from six districts of the Novosibirsk region (Dovolnoye, Barabinsk, Tatarsk, Toguchin, Bolotnoye, and Kochenyovo districts). To assess the diversity of circulating BLV genotypes, samples were collected from settlements and districts that were geographically distant from each other and had no shared pasture lands. In total, 1410 bp fragments encoding the *env* gene region were obtained from 417 BLV-positive samples. Phylogenetic analysis classified 325 BLV strains (77.9%) as genotype 4 (G4) and 92 strains (22.1%) as genotype 7 (G7). A pairwise identity matrix was constructed for 268 amino acid residues. Pairwise identity of BLV amino acid sequences in the gp51 region ranged from 96.6% to 100% for G4 and from 97.4% to 100% for G7. Multiple alignment of the amino acid sequences identified 74 mutations found in the Russian BLV variants. Through the addition of 417 novel *env* BLV sequences to GenBank, this study significantly expands the foundational data and knowledge of BLV molecular epidemiology in Russia.

## 1. Introduction

Bovine Leukemia Virus (BLV) is an oncogenic member of the genus *Deltaretrovirus* within the family *Retroviridae*. Its genome is approximately 8.7 kbp in length and shows the typical retroviral organization with *gag*, *pol*, and *env* genes flanked by long terminal repeats (LTRs). The region between *env* and the 3′ LTR encodes the regulatory proteins Tax and Rex and the accessory proteins R3 and G4, as well as viral microRNAs [1].

BLV primarily infects B lymphocytes; however, proviral DNA can also be detected in CD8+ T cells, monocytes, and granulocytes, indicating a broader leukocyte tropism than previously recognized. The natural host range of BLV includes dairy and beef cattle, but infections have also been reported in zebu and buffalo, and experimental studies demonstrate the susceptibility of sheep and goats. The virus is transmitted mainly through iatrogenic blood exposure and repeated use of contaminated instruments. Additional routes include colostrum, milk, and close contact among animals in high-density herds. Under intensive dairy production conditions, it promotes efficient horizontal spread [2].

BLV causes Enzootic Bovine Leukosis (EBL), one of the most economically significant cattle viral diseases worldwide [3]. BLV induces chronic infection through integration of its proviral DNA into the host genome, thereby establishing long-term viral persistence [4]. This chronic infection, which is often asymptomatic and observed in approximately 70% of infected animals [5], complicates early detection and leads to substantial economic losses. These losses are primarily attributable to reduced milk yield, premature culling, and trade restrictions. For instance, direct losses reached approximately USD 6,097,225 in a single Japanese region [6]. Globally, BLV prevalence can exceed 90% in some regions [7]. In Russia, an estimated 924,000 out of 15.5 million cattle are BLV carriers, ranking BLV infection among the leading causes of cattle mortality [8].

Phylogenetic analysis of the gp51 region of the BLV *env* gene is the principal method for BLV genetic classification and molecular epidemiological studies. The gp51 glycoprotein is the primary surface antigen of BLV and the principal target of neutralizing antibody responses [9]; consequently, sequence diversity in this region directly reflects immune evasion mechanisms and epidemiological transmission patterns [1]. Since the establishment of the first seven BLV genotypes in 2009 [10], substantial genetic diversity has been documented. Subsequently, genotypes 8 through 12 have been identified in geographically diverse regions, including Croatia [11], Bolivia [12], Thailand [13], Myanmar [14], China [15], and Kazakhstan [16]. The spatial distribution of BLV genotypes reveals distinct epidemiological patterns that reflect viral transmission routes and evolutionary history [15,16,17]. Consequently, the *env* gene represents an essential target for investigating BLV epidemiology. Data on the genetic diversity and geographic spread of BLV in the Russian Federation remain limited and geographically fragmented. This is particularly true for the Novosibirsk region, which harbors approximately 150.4 thousand dairy cattle in 2024 [18] and represents one of the largest cattle populations in Russia.

The Novosibirsk region is a critical dairy-producing zone in the Russian Federation and has been classified as an area with unfavorable BLV epizootic conditions [8]. However, a comprehensive molecular characterization of circulating BLV genotypes and their associated genetic diversity in this region has not been systematically documented. The identification of local BLV genotypes, intragenotype genetic variants, and biologically significant mutations is essential for understanding viral transmission dynamics, predicting immune evasion mechanisms, and designing evidence-based control strategies. The insufficient characterization of BLV molecular genetic properties in Siberian cattle populations represents a critical knowledge gap. It resulted in poor understanding of regional epidemiology and impeded the implementation of evidence-based prevention measures.

The aim of this study was to investigate the genetic diversity, phylogenetic relationships, and genotype-specific mutations of BLV circulating in dairy cattle in the Novosibirsk region by analyzing the *env* gene fragment encoding the gp51 protein.

## 2. Materials and Methods

### 2.1. Geographic Distribution of Sampling Sites

**Figure 1:**
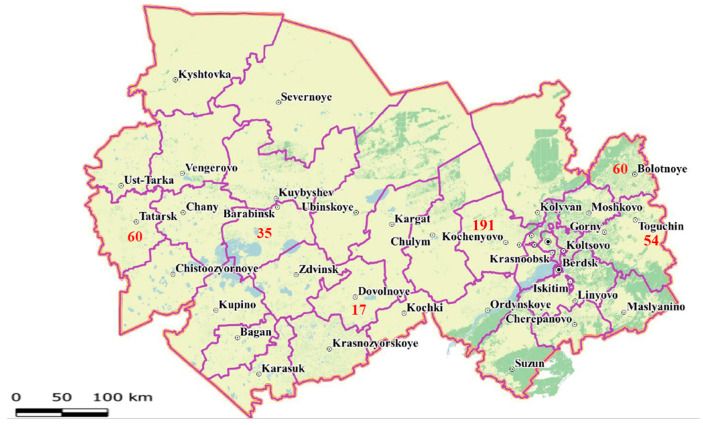
Territories of the Novosibirsk region of the Russian Federation involved in the study. The map shows the names of the large settlements of the Novosibirsk region and the number of collected cow blood samples for the 6 regions that were involved in the study.

Blood samples were collected from 417 cows that tested positive by a commercial Enzyme-Linked Immunosorbent Assay (ELISA, ID Screen^®^ BLV Competition, Innovative Diagnostics, Grabels, France; Cat. No. BLVC-10P) across six districts of the Novosibirsk region: Kochenyovo (191), Bolotnoye (60), Tatarsk (60), Toguchin (54), Barabinsk (35), and Dovolnoye (17) (Figure 1).

Farms were selected for inclusion based on accessibility and willingness to participate. All selected farms had previously identified BLV-positive animals during routine veterinary monitoring, which allowed for convenience sampling and targeted surveillance rather than strict random sampling. This study does not focus on investigating the prevalence of BLV among livestock in the Novosibirsk Region, as this requires larger-scale studies involving thousands of animals to cover ≈10% of the livestock population.

These districts were selected to represent varying climatic conditions and to ensure wide geographical separation of sampling sites.

The strategic selection of geographically distant farms (180–600 km apart) allowed for the capture of regional BLV diversity, enabling the assessment of *env* gene variation across the entire Novosibirsk region rather than within isolated districts.

The average age of the sampled animals was 4 years. The cattle belonged to the following breeds: Black-and-White Holstein, Red Steppe, Kalmyk, and Simmental. Blood samples were collected from cattle by staff of the Veterinary Directorate of the Novosibirsk Region in accordance with Russian biosafety regulations for work with pathogenic biological agents (SanPiN 3.3686-21) [19]. The study protocol was approved by the same authority. Voluntary informed consent was obtained from all cattle owners prior to BLV testing. For commercial farms, additional formal agreements for conducting research were executed.

“The study protocol was approved by Ethics Committee of State Research Center of Virology and Biotechnology “Vector” with Federal State Budgetary Educational Institution of Higher Education “Dagestan State University”, protocol code No.7 of 05.09.2025”. All animal owners provided informed consent to participate in the study.

### 2.2. Isolation of Blood Cells and DNA Extraction

Blood samples were collected from cattle via tail vein puncture using disposable sterile vacuum systems containing EDTA anticoagulant. Peripheral blood leukocytes were isolated from 250 µL of whole blood using the commercial whole blood pretreatment kit “Hemolytic” (AmpliSens, Moscow, Russia; Cat. No. 137) according to the manufacturer’s instructions. The resulting cell samples were stored at −80 °C until DNA extraction.

Genomic DNA was extracted from the mononuclear cells using a spin column method with a commercial kit "DNA extraction kit from whole blood" (DiaGene, Moscow, Russia; Cat. No. 3361.0250). DNA was eluted in 50 µL of nuclease-free water and stored at −80 °C in a freezer until PCR analysis. The concentration and purity of the extracted DNA were assessed using a NanoDrop OneC spectrophotometer (Thermo Scientific, Waltham, MA, USA; Cat. No. 13-400-519).

### 2.3. PCR Diagnostics and env Gene Fragment Sequencing

BLV DNA in the samples was detected using real-time PCR with the RealBest-Vet DNA test system for BLV (VectorBest, Novosibirsk, Russia; Cat. No. V-5441), supplemented by conventional PCR with a laboratory-designed primer set amplifying a fragment of the BLV *env* gene. The PCR to obtain a fragment of the *env* gene was custom designed to amplify a 1410 bp fragment of the BLV *env* gene encompassing the region encoding the gp51 protein. However, the lengths of the sequences deposited in GenBank under accession numbers OP850705.1–OP850797.1, ON799063.1–ON799107.1, OL660221.1–OL660401.1, and PX983326–PX983441 vary (approximately 804 to 1400 bp), because low-quality bases at the read termini were removed during post-sequencing quality control and manually trimming after multiple alignment (MUSCLE) in MEGA11 v.11.0.13 software. For one isolate (OP850778), only a 698 bp high-quality fragment of the *env* gene was retained after quality filtering, whereas for the remaining samples the deposited fragments are 804 bp or longer. PCR products were visualized by 1% agarose gel electrophoresis.

### 2.4. DNA Sequencing and Phylogenetic Analysis

Both DNA strands of the *env* gene fragments were sequenced using a 3130xl automated sequencer (Applied Biosystems, Waltham, MA, USA; Cat. No. 628-0030). The obtained sequences were edited in Sequencer v.4.1 (Gene Codes Corporation, Ann Arbor, MI, USA) and compared with reference sequences of different BLV genotypes from the GenBank international database. Our protocol successfully amplified the full-length coding region of the BLV *env* gene (~1410 bp) from all 417 BLV-positive samples. However, phylogenetic classification required standardization to ensure compatibility with reference sequences in GenBank. To standardize the analysis and exclude potential reading errors at the primer binding sites, we selected fragments of 804 nucleotides in length (excluding the leader peptide). This length provides maximum compatibility with available reference data and is consistent with internationally accepted standards for comparative analysis.

A fragment of 804 bp was selected because it spans the entire coding region of gp51 (excluding the signal peptide) and includes all major functional domains and conformational epitopes. These epitopes have commonly been used for BLV genotyping and molecular epidemiological studies in previous work. Shorter gp51 regions (e.g., 444–502 bp) have been widely employed and are sufficient to distinguish the currently recognized BLV genotypes G1–G12; however, the 804 bp fragment provides higher phylogenetic resolution while remaining directly comparable to most reference sequences in GenBank that overlap this region. Thus, the chosen fragment length maximizes compatibility with available reference data and conforms to internationally accepted standards for comparative analysis.

Preliminary analysis based on full-length ~1410 bp *env* sequences yielded the same genotype assignments (G4 and G7) and overall clustering patterns as the 804 bp alignment (Supplementary Figure S1: Phylogenetic tree for long sequences of the *env* BLV gene). This indicates the retaining of key phylogenetic signal from a shorter fragment, which is necessary for genotype classification.

In total, 445 sequences were used for the standardized alignment of the 804 bp *env* fragment. They included 417 sequences generated in this study and 28 sequences retrieved from the GenBank database. All the original sequences obtained in this study were deposited in GenBank.

Multiple alignment of the *env* gene sequences was performed with the MUSCLE algorithm in MEGA11 software [20]. The 804 bp alignment was tested for evidence of intragenic recombination using the Pairwise Homoplasy Index (PHI) test [21] as implemented in SplitsTree v.6 [22]. A *p*-value < 0.05 was considered statistically significant evidence for recombination.

Phylogenetic analysis was conducted using the Maximum Likelihood (ML) method in the IQ-TREE v1.6.12 online program [23]. The best-fit substitution model (K2P + G4) was selected using ModelFinder v.1.5.4 [24] based on the Bayesian Information Criterion. The statistical support for the phylogenetic tree topology was evaluated with 1000 bootstrap replicates.

The sequences of isolates obtained in this study were deposited in GenBank under accession numbers: OP850705.1–OP850797.1; ON799063.1–ON799107.1; OL660221.1–OL660401.1; and PX983326–PX983441.

### 2.5. Analysis of Amino Acid Variability and Selective Pressure

An 804 bp fragment covering the complete gp51 region (excluding the leader peptide) was selected to analyze amino acid variability and mutations, ensuring full coverage of all key functional domains and epitopes. Pairwise identity values for these fragments were calculated using the Sequence Demarcation Tool (SDT v1.2) software [25].

Amino acid sequences were deduced by translating the obtained nucleotide sequences using Geneious Pro v.5.3 software (https://www.geneious.com; accession date: 26 February 2026) [26].

In this study, we defined “novel mutations” as amino acid substitutions that were absent from all BLV *env* (gp51) sequences available in international databases at the time of analysis and had not been reported in previous studies on BLV genetic variability. “Russia-specific mutations” were defined as amino acid substitutions detected in our Siberian isolates and/or previously published Russian BLV sequences that were not observed in isolates from other countries. “Genotype-specific mutations” were defined as substitutions occurring exclusively within a single genotype (e.g., only in G4 or only in G7) among all analyzed sequences.

To validate the maximum parsimony results, a Bayesian inference analysis was performed using MrBayes with the GTR substitution model in Geneious Pro software (https://www.geneious.com). Identical sequences were identified and removed using the ‘Remove Redundancy’ function in Jalview v2.11.5.0 [27].

For mutation analysis, we used 78 *env* sequences of BLV strains selected after removal of identical sequences. Mutations were defined as nucleotide or amino acid differences relative to the reference strain FLK-BLV (GenBank accession number: M35242) and were recorded with their positions in the gp51 protein and assignment to known functional domains. To minimize the impact of random sequencing errors and single artifacts, only substitutions observed in at least three independent sequences were considered true variants. For each identified mutation, we documented its association with a particular genotype (G4 or G7), its functional localization (e.g., neutralizing domains ND1–ND3, epitope A, CD8+ T-cell epitope), and its frequency among all sequences analyzed.

Selection pressure on the BLV *env* gene was assessed using the codon-based Z-test of Selection implemented in MEGA 11 [20]. The Nei–Gojobori method was applied to estimate nonsynonymous (dN) and synonymous (dS) substitution rates for all pairwise sequence comparisons [28]. A dN/dS ratio (ω) significantly less than 1.0 indicates purifying selection, while ω > 1.0 suggests positive selection.

To detect site-specific episodic selection at individual codons, we applied the Mixed Effects Model of Evolution (MEME) implemented in the Datamonkey 2.0 web server using the default settings [29]. The analysis was performed on a nucleotide codon alignment of the 804 bp *env* fragment (268 codons) using the universal genetic code, with multiple nucleotide substitutions disabled (Multiple hits = None), Site Multihit set to Estimate, two rate classes (Rates = 2), no bootstrap resampling (Resample = 0), and no ancestral state reconstruction (Impute states = No). Codons with a likelihood ratio test (LRT) *p*-value ≤ 0.05 were considered to be under episodic diversifying selection.

## 3. Results

### 3.1. Phylogenetic Analysis of BLV Strains Circulating in the Novosibirsk Region

**Figure 2:**
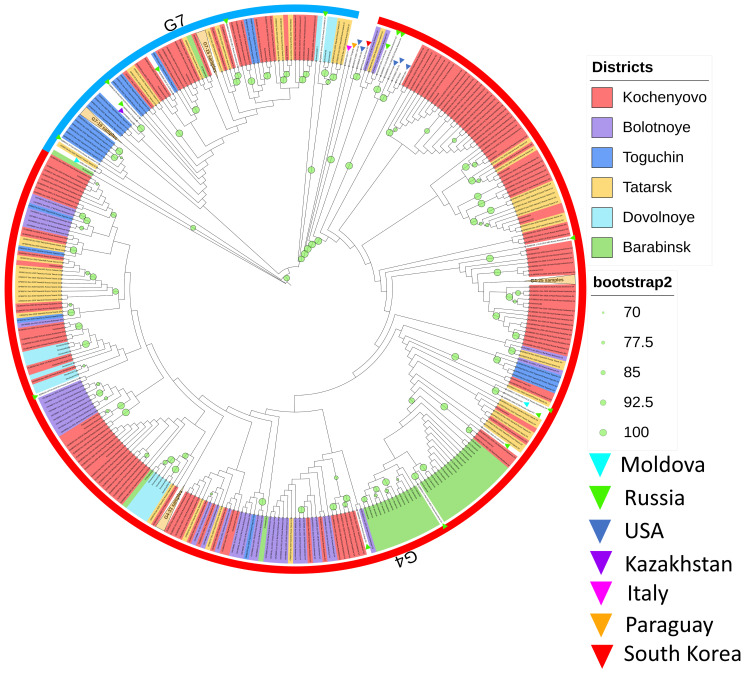
Phylogenetic analysis of an 804 bp fragment of the BLV *env* gene using the ML method. Phylogenetic analysis was conducted using the ML method (substitution model K2P + G4) implemented in the IQ-TREE v1.6.12 online program [23]. A discrete gamma distribution was applied to model evolutionary rate variation among sites. The tree is scaled, with branch lengths representing the number of substitutions per site. Branches with bootstrap support values > 70 are marked with green circles. The size of each circle corresponds to the bootstrap value (range 70–100). Sample origins are color-coded as follows: red—Kochenyovo district; yellow—Tatarsk district; purple—Bolotnoye district; light blue—Dovolnoye district; green—Barabinsk district; blue—Toguchin district. The colored branches of the tree mark the original sequences of the *env* gene obtained as part of this study (417 sequences). The black branches represent BLV reference sequences and are indicated by triangles by country and were obtained from the GenBank database (28 sequences). The sequences obtained in this study are clustered into distinct clades.

An 804-nucleotide fragment was extracted from each sequence for phylogenetic comparison with BLV *env* sequences from strains circulating worldwide. Phylogenetic analysis of the *env* gene revealed that all BLV sequences isolated in the Novosibirsk region cluster into two distinct clades corresponding to reference genotypes G4 and G7 (Figure 2). The PHI test did not detect statistically significant evidence for recombination in the 804 bp fragment (*p* = 0.1016). That confirmed the sequence region’s suitability for phylogenetic inference and that observed patterns represent genuine evolutionary divergence rather than recombination artifacts.

Consequently, the obtained sequences were classified into these two genotypes: G4 and G7. Genotype G4 appears to be predominant in the region, as it was the most prevalent variant in five of the six studied districts (Table 1) with a median value and 95% confidence interval (CI) in 84.3% (95% CI: 79.1–89.5%). The G7 genotype was dominant only in the Toguchin district, accounting for 78.3% of infected animals there.

To compare genotype frequencies between districts, we applied the chi-square test to Table 1. The analysis revealed a highly significant association between genotype and geographic origin (χ^2^ = 117.14, df = 5, *p* < 0.01), indicating that the relative frequencies of genotypes G4 and G7 differ substantially among districts. No significant association with breed or age was observed.

Isolates of the G7 genotype clustered with BLV G7 strains previously isolated in various regions of the Russian Federation, as well as in Kazakhstan and Moldova (Figure 2). The BLV G7 population examined in this study exhibited high genetic homogeneity. Phylogenetic analysis did not reveal statistically significant clustering of G7 sequences based on their farm of origin. Exceptions included minor clusters from Tatarsk (2–3 sequences, bootstrap support 74–100%), Razdolnoye (2–4 sequences, bootstrap support 90–100%), and a larger cluster from Toguchin (16 sequences, bootstrap support 74%). Furthermore, pairs or triplets of BLV G7 sequences from almost all affected farms formed phylogenetic branches with bootstrap values up to 100%, suggesting possible within-farm transmission chains.

Five of the studied BLV G7 variants formed a distinct, statistically significant cluster (Figure 2). This cluster comprised two variants from Dovolnoye and three from Tatarsk district, which are approximately 250 km apart (Figure 1). Phylogenetically, these five sequences grouped with six other G7 genotype sequences from the Russian Federation available in GenBank.

The G4 isolates from the Novosibirsk region clustered with G4 BLV strains previously reported from farms in Russia, Moldova, and the USA. Within the Kochenyovo district, the G4 genotype was detected in every farm where BLV infection was present.

The BLV study revealed multiple pairs or groups of four genetically related G4 viruses that clustered into branches with 100% bootstrap support; all isolates were obtained from cattle on the same farm. This pattern strongly supports the conclusion that BLV spreads among animals within individual farms.

The prevalence of BLV genotypes G4 and G7 differed significantly (Table 1). For the G4 genotype, infection rates on farms ranged from 24.1% to 100%, with a median of 84.3% (95% CI: 79.1–89.5%). For the G7 genotype, prevalence rates ranged from 0% to 75.9%, with a median of 15.7% (95% CI: 10.5–20.9%).

### 3.2. Analysis of Nucleotide and Amino Acid Sequences of the BLV

Analysis of nucleotide and amino acid variability was performed on the 804 bp BLV fragment beginning in the gp51 region. This region encodes the virion envelope proteins and encompasses all conformational epitopes and major functional domains of the *env* gene.

Nucleotide variability was analyzed using the 804 bp *env* BLV fragments from all 417 BLV-positive samples. For comparison, 38 *env* gene sequences spanning the same region were included from GenBank to represent all currently known BLV genotypes (G1 through G12). Sequence identity compared to genotypes G1–G12 ranged from 91.2% to 100%. Within genotypes G4 and G7, pairwise identity between our sequences and GenBank reference sequences ranged from 94.5% to 100.0%, with a median of 99.4% for G4 and from 95.6% to 100.0%, with a median of 99.6% for G7 (Supplementary Table S1: Table of identity by genotype).

The pairwise identity matrix revealed BLV sequences with 100% identity to one another. Perfect identity was most common among samples collected within the same district. However, several samples obtained from farms in geographically distant areas of the Novosibirsk region also showed 100% identity. For instance, six G4 genotype samples from Toguchin district and three G4 samples from Tatarsk district were identical, despite the approximately 600 km distance between the farms (Table 2).

Sequences with 100% nucleotide identity were excluded from the initial set of 417 based on the pairwise comparison results. Consequently, 78 unique BLV *env* gene sequences were selected for the subsequent construction of a multiple alignment using the SDT matrix and Jalview v2.11.5.0.

### 3.3. Mutation Analysis of the BLV Isolate Sequences

The 78 unique nucleotide sequences were translated into their corresponding amino acid sequences to assess mutational differences in the target BLV genomic region. Given the high degree of similarity among the nucleotide sequences (Supplementary Figure S2: The matrix of pairwise identity), these 78 variant sequences were selected for detailed analysis.

**Figure 3:**
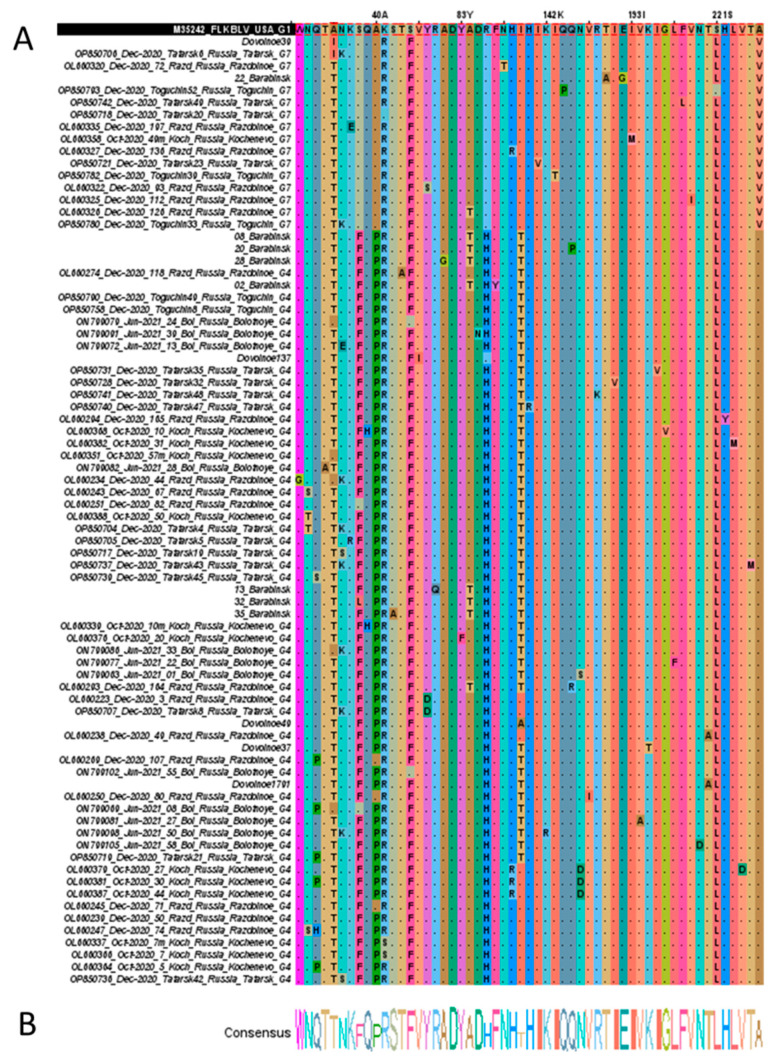
Alignment of amino acid sequences of the BLV gp51 protein, showing 78 strains isolated in the Novosibirsk region of Russia. The figure highlights differences from the FLK-BLV reference sequence: (**A**) unique *env* gene sequences after the concealment of conserved sites and removal of identical sequences in Jalview v2.11.5.0; (**B**) the consensus sequence for the multiple sequence alignment. Summary data provides a complete list of the identified mutations (Table 3).

Multiple alignment of the resulting amino acid sequences identified 74 amino acid substitutions, which comprised 44 in G4 viruses and 30 in G7 viruses (Figure 3).

Mutations present in at least two sequences were included in the analysis to exclude potential sequencing artifacts and single-occurrence variants. We described both rare single mutations (occurring in a small percentage of cases) and amino acid substitutions that were common in the majority of the studied samples (Table 3).

Compared to the FLK-BLV reference sequence (GenBank accession number: M35242), substitutions were identified at four amino acid positions common to both the G4 and G7 BLV variants.

The A15T substitution in the GP51 region was observed in 98% of BLV G4 and G7 samples. The A15I substitution was present in 13% of samples (exclusively in G7), while another 8% of samples (only in G4) encoded the reference alanine at this position.

The reference alanine at position 15 (A15) is characteristic of BLV G1 strains from the USA and G8 strains from Ukraine. The A15T substitution has been described for G4 viruses circulating in Moldova, Poland, Belgium, and France, as well as for G12 BLV. Among G7 strains from Moldova, the A15I substitution is frequently observed.

Novel, previously undescribed amino acid substitutions were identified at two positions. The K41R substitution (GP51) was present in 96% of samples. The S221L substitution (ND3) was found in 99% of samples.

The S49F substitution (GP51) was detected in 97% of samples. This substitution has been reported in G7 strains from Moldova and Russia, in G4 strains from Moldova, Africa, and France, in G8 from Ukraine, in G10 from Thailand, and in G12 from Kazakhstan.

The A258V substitution in epitope A, characteristic of G7 strains, was detected in 100% of the studied samples. This substitution has been previously reported for BLV G7 from Russia and Moldova. In the G4 sample set, the amino acid at position 258 matched the reference sequence, which is also conserved in G4 strains from Moldova, France, and Africa.

Mutations specific to Russian BLV G4 include substitutions at positions S23F (GP51), A40P (GP51), R88H (CD8+ T-cell epitope GP51), and I111T (ND1 GP51).

The S23F substitution is characteristic of G4 strains from Moldova and France. In previously studied collections, G7 viruses did not include sequences matching the reference at this position, consistent with reported G7 variants from Moldova. The A40P substitution has also been documented in G4 strains from Moldova, Belgium, France, and China.

The R88H substitution is characteristic of G4 strains from Moldova, Belgium, Poland, France, Africa, and China. Similarly, the I111T substitution has been reported in G4 sequences from Moldova, France, and Africa.

Mutations at positions N9S/T (GP51), Q10P/H/S (GP51), and N155D/S (ND2) were characteristic of two or more G4 genotype samples. In contrast, mutations A86T (CD8+ T-cell epitope) and H109R (ND1) were not specific to any particular genotype and were found in both G7 and G4.

Single mutations were identified in various regions of the *env* gene fragment. Each substitution occurring in a small percentage of cases (0.3–3%). The highest amino acid variability within our virus sample was observed in the GP51 region, particularly at position 17 (N17 → K/S/E), with the N17K substitution being the most prevalent.

Substitutions A15T, S49F, K41R, and S221L were detected in more than 97% of all analyzed sequences of genotypes G4 and G7. Thus, these changes are highly conserved within the predominant BLV genotypes circulating in the Novosibirsk region. Substitutions S23F, A40P, R88H, and I111T were observed almost exclusively in Russian G4 strains, whereas the A258V substitution in epitope A was found in 100% of G7 isolates and was absent from G4. That results support a genotype-associated distribution of these amino acid variants. The codon-based selection analysis revealed a predominance of purifying selection in *env* (mean dN/dS = 0.181 ± 0.255; 96.37% of pairwise comparisons with dN/dS < 1.0; *p* < 0.001), consistent with strong functional constraints acting on this genomic region.

Among the 268 codon positions of the BLV *env* fragment analyzed in 158 sequences, MEME (Datamonkey 2.0) identified two codon sites under episodic diversifying selection (*p* ≤ 0.05): codon 10 (*p* = 0.017) and codon 111 (*p* = 0.038). The median number of branches supporting positive selection was three per site. The remaining 266 codons (99.3%) showed no evidence of episodic positive selection at the chosen significance threshold, consistent with the overall pattern of predominant purifying selection detected by the codon-based Z-test.

## 4. Discussion

The results of the phylogenetic analysis of BLV *env* gene sequences from the Novosibirsk region indicate a close genetic relationship between the Siberian strains and virus populations circulating in European and former Soviet Union countries, including Poland, Belgium, Kazakhstan, Moldova, and Ukraine. This genetic profile aligns with previously described characteristics of the early stage of bovine leukemia spread in the Russian Federation [8].

Phylogeographic studies suggest that BLV genotype G4 was introduced into Russia mainly through the import of breeding cattle from Europe, where it may have diversified into G7 [30]. Subsequently, BLV spread to most regions of Russia, with secondary waves of introduction potentially occurring through cattle trade between Central Asian countries and Russia [31]. This multi-stage introduction history is reflected in the observed diversity of BLV genotypes in Russia, where G4 and G7 predominate [32], and recombinant forms G4/G7 are also found [33].

Analysis of 417 *env* gene sequences from BLV-positive samples obtained from six districts of the Novosibirsk region revealed that genotypes G4 and G7 are predominant in this region. Genotype G4 was classified in 325 samples (77.9%), while genotype G7 was identified in 92 samples (22.1%). This distribution is consistent with other studies that confirm G4 and G7 remain the most common BLV genotypes in the Russian Federation [34].

The geographical distribution of genotypes within the Novosibirsk region demonstrated marked unevenness. Genotype G4 predominated in five of the six studied districts (Kochenyovo, Bolotnoye, Tatarsk, Barabinsk, Dovolnoye), whereas genotype G7 was dominant exclusively in the Toguchin district. There it accounted for 75.9% of the infected animals identified. The most pronounced differences in genotype distribution were observed between the neighboring Bolotnoye district (G4: 100%, G7: 0%) and the Toguchin district (G4: 24.1%, G7: 75.9%), which are located approximately 60 km apart. This pattern may reflect the presence of local barriers to virus transmission or distinct histories of genotype introduction into individual districts.

Phylogenetic analysis revealed several distinct clusters within both genotype G4 and genotype G7. Analysis of the geographical origin of the sequences demonstrated that, in most cases, the genetically most similar BLV isolates of each genotype originated from the same farm. This finding indicates local, rather than regional, virus spread. However, clusters comprising isolates from geographically distant farms were also identified. A cluster of G7 sequences of Dolnovoye and Tatarsk is particularly noteworthy. These geographically distant districts are located approximately 250 km apart.

The identity or high similarity of sequences over such considerable geographical distances suggests that the observed pattern can hardly be explained solely by local mutation accumulation during independent virus circulation. Instead, these data may indicate a very recent common origin of these variants, linked to contemporary or recent instances of cattle trade between distant holdings. Alternatively, strong purifying selection may preserve functionally constrained regions of the BLV genome, where mutations have a significant impact on viral fitness. This could explain the observed sequence identity.

Pairwise alignment of the obtained BLV nucleotide sequences with reference sequences from GenBank revealed a high degree of nucleotide identity among the studied genotype G4 viruses, ranging from 97.3% to 100%. These values are consistent with the known characteristic of low overall genetic variability within genotype G4 compared to other BLV genotypes, which exhibit an average nucleotide distance of approximately 0.011 ± 0.002 [31,35].

For genotype G7, approximately 16 isolates formed a distinct subgroup that demonstrates reduced sequence similarity both to international reference sequences and to the majority of G7 viruses in the studied sample set. This observation could suggest either the emergence and establishment of a locally adapted G7 lineage within the Novosibirsk region or the introduction of a more divergent G7 variant from an external source. Future studies incorporating broader sets of international references could shed light on the origin and evolutionary history of this distinctive subgroup.

Comparison of the predicted amino acid sequences of the studied BLV isolates with the FLK-BLV *env* reference sequence revealed 74 amino acid substitutions characteristic of the analyzed sample set. The greatest variability was detected in the region of the *env* gene encoding the surface glycoprotein gp51, particularly within the neutralizing domain and epitope A.

The envelope signal peptide plays a crucial role in the process of incorporating the env protein into virions and directing the polypeptide chain to the endoplasmic reticulum of infected cells [36]. Data obtained from studies of HIV-1 and other retroviruses suggest that certain mutations in the signal peptide may be associated with viral infectivity and replication efficiency [37].

The neutralizing domain (ND) of the surface protein is widely recognized for its functional role in the process of viral entry into the host cell and its interaction with neutralizing antibodies, which largely determines the immunogenicity of this *env* region [31]. The I111T substitution was identified within the ND of the studied BLV sequences and found in 79% of genotype G4 samples; notably, this mutation was also detected in some genotype G7 viruses. In contrast, the A258V substitution in epitope A was identified in 100% of G7 isolates. It demonstrates high conservation specifically within the genotype G7 population and is absent in G4 samples. Thus, this pattern may indicate a genotype-specific evolutionary trajectory.

The analysis revealed a number of point mutations that were unique to individual isolates or occurred with low frequency. Several amino acid substitutions located within the critical DD’ epitopes and epitope A of the BLV genome are presumed to potentially influence viral infectivity and transmission between hosts [36]. In particular, the S253L substitution has been shown to inhibit syncytium formation while simultaneously disrupting surface glycoprotein expression [36,38].

Most of the unique substitutions identified in the Novosibirsk region samples are localized in close proximity to this critical region. This suggests that they may potentially alter the immunostimulatory and fusogenic properties of the virus, although further validation of this hypothesis through functional immunological and virological studies is required [36,38].

Previously undescribed amino acid substitutions were identified at three positions: N9 (S/T, detected in 6% of samples), Q10 (P/H/S, detected in 11% of samples), and N17 (K/S/E, detected in 10–27% of samples depending on the specific substitution). These mutations warrant further detailed investigation, as they were found in BLV isolates from animals in geographically non-overlapping districts with no clear evidence of direct contact between farms. Notably, these substitutions are not associated with any specific BLV genotype. Therefore, they may have broader evolutionary or epidemiological significance beyond individual genotypic lineages.

Notably, MEME identified codon 10 (Q10P/H/S in GP51) as being under episodic diversifying selection (*p* = 0.017). This codon belongs to a small subset of positions where mutations were detected in isolates from geographically unrelated districts. This finding suggests that selective pressure at this site is more likely driven by immune escape or functional adaptation than by random genetic drift.

Two substitutions were detected with exceptionally high frequency: K41R in the GP51 domain (96% of samples) and S221L in the ND3 domain (99% of samples). These nearly universal mutations presumably represent critical fitness determinants or functional optima established during the early stages of the historical circulation of the virus in the Siberian region of Russia.

Codon 111 (I111T in the neutralizing domain ND1) was previously identified by us as a Russia-specific G4 mutation, with a frequency of 79% among G4 isolates (*p* = 0.038). D1 is a known target of neutralizing antibodies. Detection of episodic positive selection in this region, therefore, supports the hypothesis that the substitution represents an adaptation to host immune pressure rather than a product of neutral mutation accumulation. In this context, the amino acid substitutions A15T, S49F, K41R, and S221L occurred in more than 97% of sequences from both G4 and G7. These are likely early key adaptive changes that became fixed during the initial circulation of the virus in the region and were retained across divergent genetic lineages. In contrast, the cluster of G4-specific substitutions (S23F and A40P in the GP51 domain, R88H in the CD8+ T-cell epitope, and I111T in ND1) points to a subsequent phase of local adaptation that likely arose during the divergence and establishment of genotype G4 in the Siberian cattle population. These substitutions occur at frequencies of 95–98% in G4 samples and are almost absent from G7, underscoring their putative genotype-specific nature.

The G7-specific A258V substitution in epitope A, identified in 100% of G7 isolates and completely absent in G4 samples, demonstrates a clearly defined genotype-specific evolutionary trajectory, reflecting the different adaptive histories and ecological niches of these two major BLV lineages in the Novosibirsk region.

Codon-based selection analysis of contemporary BLV populations in the Novosibirsk region revealed a clear predominance of purifying selection (mean dN/dS = 0.181 ± 0.255; 96.37% of pairwise comparisons with dN/dS < 1.0; *p* < 0.001). It indicates that most historically accumulated mutations have become fixed and are maintained by negative selection acting to preserve the functional integrity of env proteins. Against this background, MEME (Datamonkey 2.0) identified only two codon sites under episodic diversifying selection among the 268 positions examined: codons 10 (*p* ≈ 0.017) and 111 (*p* ≈ 0.038), with a median of three branches per site supporting positive selection. The remaining 266 codons (99.3%) showed no evidence of episodic positive selection, consistent with an overall pattern of evolutionary stasis and constrained nucleotide substitution in Siberian BLV populations. Thus, gp51 is predominantly under a purifying selection regime and is highly conserved. Only a few sites in functionally important domains appear to experience episodic diversifying selection, which likely reflects localized episodes of adaptation to host immune pressure [39].

The biologically significant mutations identified in this study within the BLV *env* gene regions encoding the GP51 domains, the CD8+ T-cell epitope, ND1, ND3, and epitope A provide valuable information for developing virus control strategies. The concentration of the majority of mutations (11 out of 19 major variations) specifically in the GP51 domain, which is the main immunogenic site of the virus, suggests that these substitutions may represent viral adaptations aimed at escaping immune pressure from the host [38]. However, this hypothesis requires further validation through functional immunological and virological studies.

Although this study provides the most extensive molecular characterization to date of the BLV *env* gene in the Novosibirsk region, several limitations should be acknowledged. First, our analysis focused exclusively on *env*. Although it is highly informative for genotyping and phylogenetic inference, it does not capture the full genomic diversity of BLV or potential recombination events in other genomic regions. Second, the limited number and heterogeneous quality of full-length *env* sequences from neighboring regions available in public databases constrained our ability to perform more detailed phylogeographic reconstructions. We also did not perform a formal phylogeographic analysis (e.g., using Bayesian diffusion models); therefore, our hypotheses regarding possible BLV dissemination routes remain preliminary and should not be interpreted as definitive. Despite these limitations, the large dataset generated here, comprising 417 new *env* sequences, substantially expands the global BLV sequence repository and provides a robust foundation for future work. Follow-up studies incorporating whole-genome sequencing and broader geographic sampling will be required to refine the evolutionary history, transmission pathways, and adaptive mechanisms of BLV circulating in Siberia and beyond.

In summary, this study provides the most comprehensive molecular characterization of the BLV *env* gene in the Novosibirsk region to date, revealing the co-circulation of genotypes G4 and G7 with a distinct geographical distribution. Phylogenetic analysis confirmed the genetic relatedness of Siberian strains to European and Central Asian isolates, supporting historical introduction routes and subsequent local diversification. The identification of 74 amino acid substitutions, including several novel and genotype-specific mutations (e.g., S23F, A40P, R88H, I111T in G4; A258V in G7), highlights the ongoing evolutionary dynamics of BLV within regional cattle populations. Notably, the high frequency of mutations within the immunodominant gp51 domain suggests potential adaptive mechanisms for immune evasion. However, it warrants further functional validation through in vitro and in vivo studies. The predominance of purifying selection indicates that most observed mutations have become fixed and are now maintained to preserve viral fitness. These findings not only significantly expand the global BLV sequence database with the addition of 417 *env* sequences but also provide a critical foundation for future research directions. These include functional characterization of novel mutations to assess their impact on viral infectivity, antigenicity, and transmission and rational design of region-specific vaccine candidates incorporating conserved and high-frequency epitopes. Ultimately, this work underscores the need for continuous molecular surveillance to monitor viral evolution and inform evidence-based control strategies tailored to the epidemiological landscape of Siberia and other BLV-affected regions.

## 5. Conclusions

This study clarifies distribution, diversity, and evolutionary dynamics of BLV in the Novosibirsk region—a major dairy-producing region of Russia. Detection of two predominant genotypes—G4 and G7—coupled with identification of 74 novel amino acid substitutions in the *env* gene provides essential data on BLV evolution in Siberian cattle populations.

Sequencing was performed on 417 *env* gene fragments obtained from a strategically distributed set of BLV-positive samples collected from cattle across six districts of the Novosibirsk region. Analysis identified several Russia-endemic amino acid substitutions, including S23F, A40P, R88H, and I111T within genotype G4 and A258V within genotype G7. The functional relevance and biological significance of these mutations remain to be determined through further functional and molecular studies. Together, these findings reveal distinct phylogenetic clustering and inter-district transmission dynamics that are critical for developing targeted control strategies.

The deposit of 417 new *env* gene sequences into GenBank significantly expands the available genetic data on BLV in Russia and enables more robust comparative and phylogenetic analyses both within the Russian Federation and internationally. These data establish a foundation for developing enhanced diagnostics, designing vaccine candidates effective against circulating genotypes and shaping strategic control programs suitable for both large-scale operations and small private farms. Collectively, this work advances understanding of BLV genetics and provides essential information for improving livestock health and economic security in Russia and other affected regions.

## Figures and Tables

**Table 1:** Distribution of BLV Genotypes G4 and G7 in Districts of the Novosibirsk Region.

Origin of Sample	Number of Sequences	Breed	Age (y) M ± SD	Genotype G4 95% CI	Genotypes G7 95% CI
Dovolnoye district (Dov)	17	Steppe	4.3 ± 0.7	11 (64.7%) (95% CI: 42.0–87.4%)	6 (35.3%) (95% CI: 12.6–58.0%)
Barabinsk district (Bar)	35	Black and White	4.7 ± 2.4	33 (94.3%) (95% CI: 86.6–100.0%)	2 (5.7%) (95% CI: 0.0–13.4%)
Tatarsk district (Tat)	60	Steppe, Black and White	4.5 ± 0.5	42 (70%) (95% CI: 58.4–81.6%)	18 (30%) (95% CI: 18.4–41.6%)
Toguchin district (Tog)	54	Simmental, Black and White	3.8 ± 0.8	13 (24.1%) (95% CI: 12.7–35.5%)	41 (75.9%) (95% CI: 64.5–87.3%)
Bolotnoye district (Bol)	60	Steppe, Black and White	4.6 ± 1.1	60 (100%) (95% CI: 100.0–100.0%)	0 (95% CI: 0.0–0.0%)
Kochenyovo district (Koch, Razd)	191	Black and White, Steppe, Simmental	7.0 ± 5.0	161 (84.3%) (95% CI: 79.1–89.5%)	30 (15.7%) (95% CI: 10.5–20.9%)

**Table 2:** Clustering Analysis Based on Pairwise Identity Values of BLV Strains from Different Districts of the Novosibirsk Region.

	Kochenyovo (*N* = 191)	Bolotnoye (*N* = 55)	Tatarsk (*N* = 54)	Toguchin (*N* = 45)	Dovolnoe (*N* = 17)	Barabinsk (*N* = 35)
Kochenyovo (*N* = 191)	G4:9, G7:8					
Bolotnoye (*N* = 55)	G4:14, G7:0	G4:13, G7:0				
Tatarsk (*N* = 54)	G4:6, G7:4	G4:16, G7:0	G4:7, G7:8			
Toguchin (*N* = 45)	G4:0, G7:2	G4:16, G7:0	G4:9, G7:0	G4:0, G7:3		
Dovolnoe (*N* = 17)	G4:2, G7:0	G4:0, G7:0	G4:0, G7:0	G4:0, G7:0	G4:5, G7:0	
Barabinsk (*N* = 35)	G4:5, G7:0	G4:0, G7:0	G4:0, G7:0	G4:0, G7:0	G4:0, G7:0	G4:7, G7:0

**Table 3:** The main mutations of the *env* BLV gene sequences from the Novosibirsk region with information on the localization of the mutation in a separate domain, genotype and prevalence of the mutation among all analyzed sequences.

Mutation	Functional Domain	Prevalence	Genotypes
N9S/T	GP51	6%	G4
Q10P/H/S	GP51	11%	G4
A15T	GP51	98%	G4, G7
A15I	GP51	13%	G7
N17K	GP51	10%	G4, G7
N17S/E	GP51	5%	G4
S23F	GP51	Russian G4-specific (97%)	G4
A40P	GP51	Russian G4-specific (95%)	G4
K41R	GP51	96%	G4, G7
K41S	GP51	3%	G4
S49F	GP51	97%	G4, G7
A86T	CD8+ T-cell Epitope	12%	G4, G7
R88H	CD8+ T-cell Epitope	Russian G4-specific (98%)	G4
H109R	ND1 (Neutralizing Domain 1)	5%	G4, G7
I111T	ND1 (Neutralizing Domain 1)	Russian G4-specific (79%)	G4
N155D/S	ND2 (Neutralizing Domain 1)	6%	G4
S221L	ND3 (Neutralizing Domain 3)	99%	G4, G7
A258V	Epitope A	Russian G7-specific (100%)	G7
Additional substitutions	Various domains	1.2–3.8%	G4, G7

## Data Availability

The nucleotide sequences of the BLV *env* gene generated in this study have been deposited in the GenBank database under accession numbers: OP850705.1–OP850797.1; ON799063.1–ON799107.1; OL660221.1–OL660401.1; PX983326–PX983441.

## Supplementary Materials

The following supporting information can be downloaded at: https://www.mdpi.com/article/10.3390/pathogens15040405/s1. The following supporting information is available: Supplementary Figure S1: Phylogenetic tree for long sequences of the *env* BLV gene: additional phylogenetic tree using longer *env* gene fragments. Supplementary Figure S2: The matrix of pairwise identity: a matrix of pairwise nucleotide sequence identity of the obtained fragments, demonstrating a high level of homology. Supplementary Table S1: Table of identity by genotype: a table with quantitative information (%) about the level of identity between the obtained nucleotide sequences of the *env* gene and the reference sequences.

## Abbreviations

The following abbreviations are used in this manuscript:

BLV	Bovine Leukemia Virus
FLK	Fetal Lamb Kidney
EBL	Enzootic bovine leukosis
ELISA	Enzyme-Linked Immunosorbent Assay
G4	Genotype 4
G7	Genotype 7
PCR	Polymerase Chain Reaction
ML	Maximum Likelihood
CI	Confidence Interval
PHI	Pairwise Homoplasy Index
SDT	Sequence Demarcation Tool
LLC	Limited Liability Company
EDTA	Ethylenediaminetetraacetic acid
BLAST v.2.17.0	Basic Local Alignment Search Tool
MUSCLE	Multiple Sequence Comparison by Log-Expectation
BIC	Bayesian Information Criterion
GTR	General Time Reversible (model)
dN	Nonsynonymous substitution rate
dS	Synonymous substitution rate
ND	Neutralizing Domain
GP51	Surface glycoprotein 51 (gp51)
HIV-1	Human Immunodeficiency Virus type 1
SanPiN	Sanitary Rules and Norms
GBU NSO	Government Budgetary Institution of Novosibirsk Oblast
NGS	Next-Generation Sequencing
MEME	Mixed Effects Model of Evolution
LRT	Likelihood Ratio Test
LTR	Long Terminal Repeat
SD	Standard Deviation
