# Supplementary figures and images for: Genetic Diversity of the BLV env Gene and gp51 Mutations in Genotypes G4 and G7 Circulating in Dairy Cattle in the Novosibirsk Region (Western Siberia, Russia)
Source: Pathogens. 2026 Apr 8;15(4):405. doi: 10.3390/pathogens15040405 (PMC13118777; doi:10.3390/pathogens15040405)

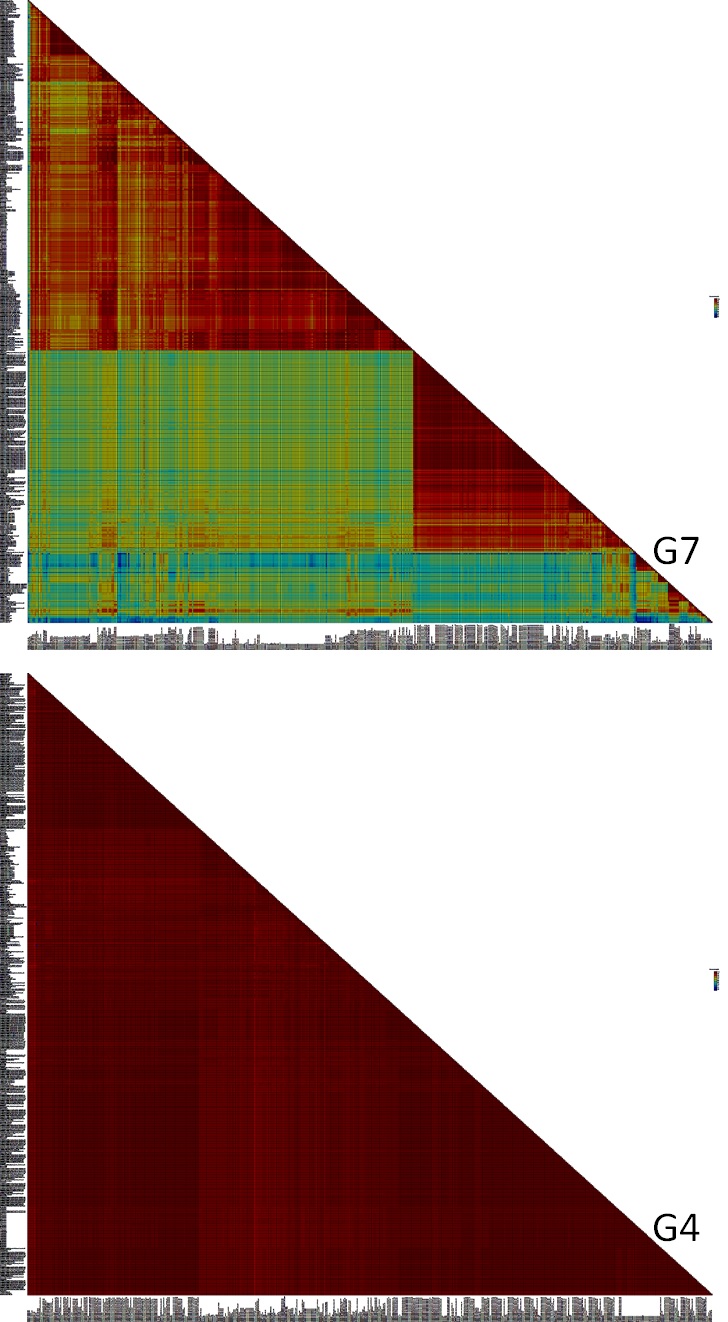

Supplement: Supplementary file 1 [file pathogens-15-00405-s001.zip › Supplementary Figure S2 The matrix of pairwise identity.jpg]
